# Contributions of Three Starch Branching Enzyme Isozymes to the Fine Structure of Amylopectin in Rice Endosperm

**DOI:** 10.3389/fpls.2018.01536

**Published:** 2018-10-23

**Authors:** Takayuki Sawada, Mizuho Itoh, Yasunori Nakamura

**Affiliations:** ^1^Faculty of Bioresource Sciences, Akita Prefectural University, Akita, Japan; ^2^Akita Natural Science Laboratory, Akita, Japan

**Keywords:** amylopectin, chain-length distribution, endosperm, rice, starch, starch biosynthesis, starch branching enzyme

## Abstract

Three starch branching enzyme (BE) isozymes, BEI, BEIIa, and BEIIb, are involved in starch biosynthesis in rice endosperm. Past *in vivo* and *in vitro* studies have suggested that each BE isozyme plays a distinct role in forming the fine structure of amylopectin. To elucidate more details of their roles, we prepared DNA constructs in which all the possible combinations of the expressions of these three isozymes were suppressed in developing rice endosperm. Analysis of the chain-length distributions of amylopectin produced under these various conditions confirmed the contributions of the individual BE isozymes to the fine structure of amylopectin in rice endosperm. Among these isozymes, the impact of loss of BEIIb activity on amylopectin fine structure was most remarkable and indicated that it plays a specific role in the synthesis of short chains with a 6–13 degree of polymerization (DP). The contribution of BEI to the amylopectin synthesis was unclear when only BEI activity was reduced. It was clear, however, when both BEI and BEIIb activities were substantially inhibited. The DP11-22 intermediate chains were markedly reduced in the Δ*BEI/BEIIb* line compared with the Δ*BEIIb* line, indicating that BEI plays a distinct role in the synthesis of these intermediate chains. Although no substantial change in amylopectin chain profile was detected in the Δ*BEIIa* line, the role of BEIIa could be deciphered by analyzing amylopectin fine structure from the Δ*BEI/BEIIa/BEIIb* line in comparison to that from Δ*BEI/BEIIb* line. This strongly suggests that BEIIa compensates for the role of BEI, rather than that of BEIIb, by forming intermediate chains of DP11-22. In addition, the new possibility that BEIIa is involved in the formation of starch granules in rice endosperm was suggested because the onset temperature for gelatinization of starch granules in the Δ*BEIIa/BEIIb* line was significantly higher than that in the Δ*BEIIb* line. In summary, the present study highlights the distinct roles of BEI, BEIIa, and BEIIb in the synthesis of amylopectin in developing rice endosperm.

## Introduction

Starch branching enzyme (BE) is the only enzyme capable of forming the branch linkages in amylopectin, a major starch component, that usually comprises 65–85% of starch. BEs in higher plants belong to glycoside hydrolase family 13 (GH13) in the Carbohydrate-Active Enzymes Database (CAZy; Stam et al., 2006), and are further classified into two types: BEI and BEII (Boyer and Preiss, 1978; also see the review by Preiss and Levi, 1980). In addition, cereals contain BEIIa- and BEIIb-type isozymes, the latter being specifically expressed in their endosperms. The functional properties of BEI, BEIIa, and BEIIb were at first extensively studied using maize plants, mainly by Preiss and his colleagues. They showed that BEI plays a role in the formation of the intermediate and long chains of amylopectin whereas BEIIa and BEIIb preferentially form its short chains in maize endosperm (Guan and Preiss, 1993; Takeda et al., 1993; Guan et al., 1997). Later, by using high-performance anion-exchange chromatography- pulsed amperometric detection (HPAEC-PAD) or fluorophore-assisted carbohydrate electrophoresis (FACE), the contribution of each BE isozyme to the fine structure of amylopectin was analyzed by precisely measuring the chain-length distribution of amylopectin formed in mutants and transformants in which the activities of BE isozymes were singly or multiply modified (see the review by Nakamura, 2015, 2018; Tetlow and Emes, 2017). In these analyses, the lengths of α-1,4 chains [i.e., their degree of polymerization (DP) values] liberated after debranching the α-1,6 glucosidic linkages (branches) of amylopectin with isoamylase (ISA) were measured by the FACE method. Chain-distribution analysis of glucans formed by *in vitro* BE enzymatic reactions also confirmed the chain-length specificity of each isozyme and characterized its enzymatic properties, such as substrate specificity toward branched and linear glucans and malto-oligosaccharides (Nakamura et al., 2010; Sawada et al., 2014; also see the review by Nakamura, 2015).

The roles of individual BE isozymes in starch biosynthesis in rice endosperm have been examined by many groups worldwide using mutants and transformants prepared from both *japonica*-type and *indica*-type rice cultivars. It has been reported that although the relative activities of BEI, BEIIa, and BEIIb in both cultivars are reported approximately 60–80%, 10–20%, and 10–20%, respectively (Yamanouchi and Nakamura, 1992), the impacts of the three BE isozymes on the starch synthesis and structure greatly differ among them (Nakamura, 2002). Mutations in the *BEIIb* gene resulted in the *amylose-extender* (*ae*) phenotype of the caryopses, which had a floury appearance and reduced weight (Mizuno et al., 1993; Nishi et al., 2001). The *ae* mutant starch contained modified amylopectin with fewer short chains of with DP ≤ 17 (mostly A chains) and more long B chains (see Supplementary Figure S1) and an elevated amylose content, which caused changes to the starch granular structure and physicochemical properties, such as a switch in X-ray diffraction pattern from A-type to B-type and a higher resistance to thermal gelatinization (Nishi et al., 2001). These results are consistent with those of Butardo et al. (2011), who showed that RNA silencing of *BEIIb* expression in rice kernels led to the *ae* phenotypes similar to those described above. Elimination of BEI activity led to only slight phenotypic changes in amylopectin structure, such as the elevation of short chains and a reduction in long B chains, while no significant change in the appearance and weight of the caryopsis were found (Satoh et al., 2003). No detectable alterations in the amylopectin chain profile and caryopsis phenotypes were detected when the *BEIIa* gene was defective (Nakamura, 2002). These results are basically consistent with the view proposed by the Preiss’ group that BEI and BEIIb play crucial roles in the formation of short and long chains of amylopectin in maize endosperm (Guan and Preiss, 1993; Takeda et al., 1993; Guan et al., 1997).

Biochemical, molecular biological, and genetic approaches using additional mutants or transformants in which two or three BE isozymes are simultaneously eliminated would be very useful in precisely defining the contributions of the three BE isozymes to amylopectin biosynthesis. Wei and his group have extensively studied the starch phenotypes of mainly the *be1*/*be2b* mutants of both *japonica*- and *indica*-type rice lines, thereby revealing the roles of both BEs in amylopectin fine structure, amylose content, and starch structure, and starch functional properties including resistance to enzyme-catalyzed hydrolysis (Wei et al., 2010; Man et al., 2012, 2013, 2014; Wang et al., 2018; see also the review by Wang et al., 2017). However, the contribution of BEIIa in starch biosynthesis is still uncertain, perhaps because its role overlaps that of BEIIb and/or BEI to large extent. In this study, we prepared transformed lines in which all the possible combinations of BE isozymes were eliminated by RNAi technology, namely the seven lines: Δ*BEI*, Δ*BEIIa*, Δ*BEIIb*, Δ*BEI/BEIIa*, Δ*BEI/BEIIb*, Δ*BEIIa/BEIIb*, and Δ*BEI/BEIIa/BEIIb*. By comparing the starch phenotypes among these lines, with a particular emphasis on precisely analyzing amylopectin chain-length distributions by the FACE method (O’Shea et al., 1998), the contributions of the three BE isozymes to amylopectin biosynthesis in rice endosperm have been clarified, and in particular the role of BEIIa on the amylopectin structure and the starch gelatinization properties.

## Materials and Methods

### Preparation of cDNA Fragments for Rice BE Isozymes

The cDNA fragments of rice BEI, BEIIa, and BEIIb used for preparation of RNAi constructs were generated by PCR using cDNA prepared from mRNA of developing seeds of the *japonica*-type rice cultivar Kinmaze, as described previously (Nakamura et al., 2005). The forward and reverse primers used were: BEI, 5′- ggggacaagtttgtacaaaaaagcaggctATGCTGTGTCTCACCTCCTCTTCCTCCTC-3′ and 5′- ggggaccactttgtacaagaaagctgggtATATATAGGAAGGTGGTCGACCTCCTCCAC-3′; BEIIa, 5′- ggggacaagtttgtacaaaaaagcaggctGCCGTCGGTGCTCTTCAGGAGGAAGGACTCC -3′ and 5′- ggggaccactttgtacaagaaagctgggtTGCCACTGCTGGAATCTCTTCCTCCTCCTC -3′; BEIIb, 5′- ggggacaagtttgtacaaaaaagcaggctACGGGATGCCGGTTTCAGCAGGTTCAGACG -3′ and 5′- ggggaccactttgtacaagaaagctgggtCTGTTGGTGGGACAACTCGTGGTTTCTGC -3′ (Note that small and large letters represent tags and coding regions of *BE* genes, respectively). The PCR reaction products were separated by agarose gel (1.5% agarose, w/v) electrophoresis and the corresponding bands were excised from the gels.

For the preparation of cDNA fragments of *BEI* plus *BEIIa* cDNA by PCR, the forward primer used for preparation of the BEI cDNA (see above), the reverse primer used for preparation of the BEIIa cDNA (see above) and a BEI-BEIIa connection primer (5′- TTGTGGAGGAGGTCGACCACCTTCCTATATATgccgtcggtgctcttcaggaggaaggac-3′) were used. The cDNA fragments of *BEI* and *BEIIa* that were used for the PCR were prepared using 5′-ATGCTGTGTCTCACCTCCTCTTCCTCCTC-3′ and 5′- ATATATAGGAAGGTGGTCGACCTCCTCCAC-3′, and 5′-GCCGTCGGTGCTCTTCAGGAGGAAGGACTCC-3′ and 5′-TGCCACTGCTGGAATCTCTTCCTCCTCCTC-3′, respectively. PCR reaction products were separated by agarose gel (1.5% agarose, w/v) electrophoresis and the corresponding bands were excised from the gel and used for the subsequent reaction.

For preparation of cDNA fragments of *BEI* and *BEIIb* cDNA by PCR, the forward primer used for preparation of the BEI cDNA (see above), the reverse primer used for preparation of the BEIIb cDNA (see above) and the BEI-BEIIb connection primer (5′- GTTGTGGAGGAGGTCGACCACCTTCCTATATATacgggatgccggtttcagcaggttcag-3) were used. The cDNA fragment of *BEI* used for PCR was prepared as above, and that of *BEIIb* was prepared by using 5′-ACGGGATGCCGGTTTCAGCAGGTTCAGACG-3′ and 5′-CTGTTGGTGGGACAACTCGTGGTTTCTGC-3′. PCR reaction products were separated by agarose gel (1.5% agarose, w/v) electrophoresis and the corresponding bands were excised from the gel and used for the subsequent reaction.

For preparation of cDNA fragments of *BEIIa* and *BEIIb* cDNA by PCR, the forward primer used for preparation of the BEIIa cDNA (see above), the reverse primer used for preparation of the BEIIb cDNA (see above) and the BEIIa-BEIIb connection primer (5′- TCTGAACCTGCTGAAACCGGCATCCCGTtgccactgctggaatctcttcctcctcctc-3′) were used. The cDNA fragments of *BEIIa* and *BEIIb* that were used for the PCR were prepared. The DNA fragments were purified with an agarose gel, as described above.

For preparation of cDNA fragments for *BEI*, *BEIIa*, and *BEIIb* cDNA by PCR, the forward primer used for preparation of the BEI cDNA (see above), the reverse primer used for preparation of the BEIIb cDNA (see above) and the BEIIa-BEIIb connection primers (see above) were used. The cDNA fragments of *BEI* plus *BEIIa* and *BEIIb* used as templates for the PCR were prepared as above, and DNA fragments were purified from agarose gels, as described above.

### Preparation of DNA Constructs for Silencing the Expression of Rice *BE Isozyme* Genes

**FIGURE 1:**
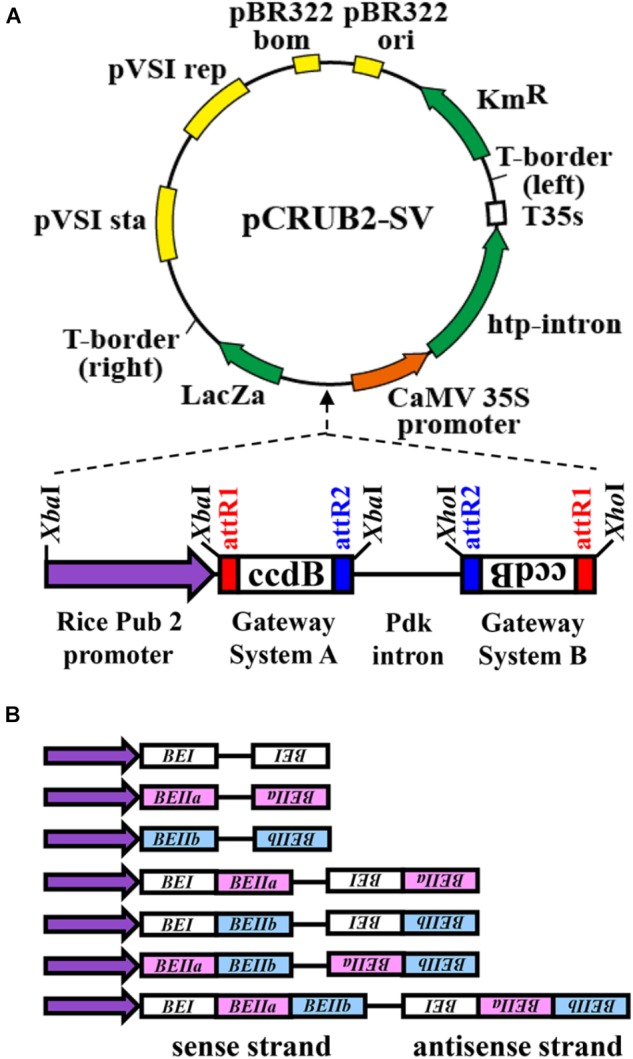
Representation of DNA constructs used for RNAi-suppression of *BEI*, *BEIIa*, and *BEIIb* gene expression in rice endosperm (Helliwell et al., 2002). **(A)** The rice polyubiquitin 2 promoter (rice Pub2 promoter) was used and attR1 and attR2 are recombination recognition sites of the target gene. The ccdB protein selectively inhibits *Escherichia coli* DNA topoisomerase type II and inhibits the growth of most *E. coli* strains such as OmniMAX^TM^ 2-T1R, DH5α^TM^, TOP10. This was used for selection for recombination of the target gene. The pdk intron was used to construct the hairpin loop RNA of the RNAi. The CaMV 35S-driven hygromycin resistance gene (hpt intron) was used for selection of transgenic plants. **(B)** The DNA fragments for the target *BE* genes used for the seven different RNAi.

The RNAi constructs were designed by including oligonucleotides for cDNA fragments encoding rice BEI, BEIIa, and BEIIb, respectively, as illustrated in Figure 1. The binary vector pINDEX 4 (Ouwerkerk et al., 2001) was used as the basic plasmid DNA for the RNAi construct. The rice polyubiquitin 2 promoter (Wang et al., 2000), Gateway system fragments A and B, and the pdk intron fragment were prepared using the pHELLSGATE 8 vector (Helliwell et al., 2002; Figure 1). For preparation of the rice polyubiquitin 2 promoter fragment by PCR, 5′-atgtctagaCTGCAGAAATGCAAATTTCATAAAAC-3′ and 5′-atgtctagaCTCGAGGGTGATAGTCTTGCCGGTC-3′ were used as forward and reverse primers, respectively. An *Xba*I site was added to both ends of the fragment during this PCR. The fragment was then prepared by restriction enzyme treatment with *Xba*I, while the pHELLSGATE 8 vector was treated with *Xba*I and *Xho*I to prepare three fragments; the Gateway system fragments A and B and pdk intron fragment (*Xba*I–*Xho*I). These four fragments were introduced into the binary vector pINDEX 4, which had been digested with *Xba*I and *Xho*I. The final plasmid construct was designated as pCRUBQ-SV. Seven kinds of cDNA fragments for BEs were then inserted into the pCRUBQ-SV plasmid using the Gateway system (Katzen, 2007).

### Generation and Selection of Transgenic Plants

A total of seven different DNA constructs were used to transform competent *Agrobacterium tumefaciens* EHA105 cells (Hood et al., 1993). Transformants were selected on LB-agar plates containing 50 μg/ml kanamycin.

Calli of *japonica*-type rice cultivar Kinmaze were generated from sterile seeds and transformed with recombinant *A. tumefaciens* EHA105 carrying the plasmid pCRUB2-SV/ΔBEs. Selection of transformants and callus regeneration were as described previously (Sawada et al., 2009).

For each construct, a total of about 30 independent T_0_ progeny lines were grown in a greenhouse which was controlled at 30°C and 60% relative humidity. One to three developing T_1_ seeds were randomly chosen. Crude enzyme extracts were prepared from each kernel and separated by native polyacrylamide gel electrophoresis (PAGE) to detect BE activities (see below). Six to nine lines were then selected for every construct, in which BE activities were judged as having been greatly inhibited as expected, and seeds from these lines were further analyzed.

### Preparation of Enzyme Extracts and Native-PAGE of BE Isozymes From Developing Rice Endosperm

A single developing kernel at the mid-milking stage was homogenized using a plastic pestle in a microtube on ice in 100 μl of 50 mM imidazole-HCl (pH 7.4), 8 mM MgCl_2_, 50 mM 2-mercaptoethanol, and 12.5% (v/v) glycerol. The homogenate was centrifuged at 10,000 *g* at 4°C for 20 min, and the supernatant was used as the crude enzyme extract. Ten μl of each extract was applied onto a native-polyacrylamide gel and native PAGE/staining of BE isozyme activity was performed as described previously (Yamanouchi and Nakamura, 1992). Native PAGE was performed on a slab gel prepared of 7.5% (w/v) resolving gel with 3.3% (w/v) stacking gel using a modified version of the method described by Davis (1964). Electrophoresis was carried out at 4°C at a constant current of 20 mA. After electrophoresis, the gel was rinsed with 20 ml of a solution of 50 mM HEPES-NaOH buffer (pH 7.4) containing 20% (v/v) glycerol for 2–3 min on ice and then incubated for 5–6 h at 30°C in 20 ml of the BE reaction mixture, which consisted of 50 mM HEPES-NaOH buffer (pH 7.4), 50 mM glucose 1-phosphate, 2.5 mM AMP, 10% (v/v) glycerol, and rabbit muscle phosphorylase a (Pho-a; about 60 units; Sigma, St. Louis, MO, United States). The gel was then placed in a solution of 0.1% (w/v) I_2_ and 1% (w/v) KI. BE activity bands were detected by their reddish purple color due to the formation of branched glucans by BE and Pho-a or the plastidial phosphorylase (Pho1). Although BEIIb and Pho1 bands overlapped, while Pho1 activity itself was visualized as a blue band from the presence of linear glucans when BEIIb activity was absent, as described previously (Yamanouchi and Nakamura, 1992).

### Observation of Rice Kernel Morphology

Cross-sections were prepared from the middle part of mature caryopses using a razor blade and images were captured with a digital camera under suitable lighting.

### Measurements of Mature Kernel Weights

The weight of a mature kernels was determined as the average value of 15 arbitrarily chosen mature caryopses.

### Thermal Properties of Starch Granules

Thermal properties of starch granules were analyzed using a differential scanning calorimeter (DSC), as described previously (Fujita et al., 2003). For the DSC measurement of thermal properties of endosperm starch, rice powder prepared from a half portion of each kernel by de-hulling, crushing with pliers, and hand-homogenization using a mortar and pestle. The weighed starch (about 3 mg) was placed in an aluminum sample cup (SSC000C009; Seiko Instrument, Tokyo, Japan), mixed with 9 μl of distilled water and sealed. Gelatinization properties of the starch were analyzed by DSC-6100 (Seiko Instrument, Tokyo, Japan). The heating rate was 3°C per min in a temperature range of 5–90°C.

### Analysis of Chain-Length Distribution of Amylopectin

The chain-length distribution of amylopectin was determined by the FACE method (O’Shea et al., 1998), as described by Nakamura et al. (2002). Starch samples from the other half of the kernel remaining after analysis of starch thermal properties by DSC, as described above, were homogenized in a mortar and pestle. The powder was suspended in 5 ml of methanol and heated in a boiling water bath for 10 min. The suspension was centrifuged at 2,000 *g* for 5 min, and the precipitate was washed twice with 5 ml of 90% (v/v) methanol and centrifuged again at 2,000 *g* for 5 min. The supernatant was removed and the precipitate was dried in air. The rice powder (1 mg) was suspended in 1 ml of distilled water and heated in a boiling water bath for 5 min to gelatinize starch. An aliquot (100 μl) was placed into a microtube and added by 4 μl of 100% acetic acid, 4 μl of 600 mM Na-acetate buffer (pH 4.4), and 91 μl of distilled water. To debranch the amylopectin, 1 μl of a *Pseudomonas amyloderamosa* ISA solution (PaISA; about 59 units; Hayashibara Biochemical Laboratories, Inc., Okayama, Japan) was added and the sample was incubated at 37°C for 3 h. The debranched sample was heated in a boiling water bath for 5 min to stop the enzymatic reaction. The solution was then deionized with ion exchange resin [15 mg; AG 501-X8(D) Resin; Bio-Rad Laboratories, Inc.] for 30 min by rotating the microtube at 10 rpm at room temperature. Fluorescence labeling of α-1,4-glucan chains at their reducing ends with 8-amino-1,3,6-pyrenetrisulfonic acid (APTS) was performed using an APTS Labeling Dye kit according to the instruction manual (SCIEX; Tokyo, Japan). The APTS-labeled α-1,4-glucans were analyzed using a capillary electrophoresis system equipped with a laser-induced fluorescence detector (P/ACE MDQ Carbohydrate System; Beckman Instruments/AB SCIEX, Tokyo, Japan).

## Results and Discussion

### DNA Construct for Suppression of the *BE Isozyme* Gene Expression

In the present investigation, gene expression of BEI, BEIIa, and/or BEIIb was selectively suppressed by using RNAi constructs containing specific regions of cDNA coding for BE isozymes. The lengths of regions used for BEI, BEIIa, and BEIIb cDNA were 237 bp (coding region, 1–237; Accession number, D10752), 220 bp (115–324; AB023498), and 183 bp (242–424; D16201), respectively.

**FIGURE 2:**
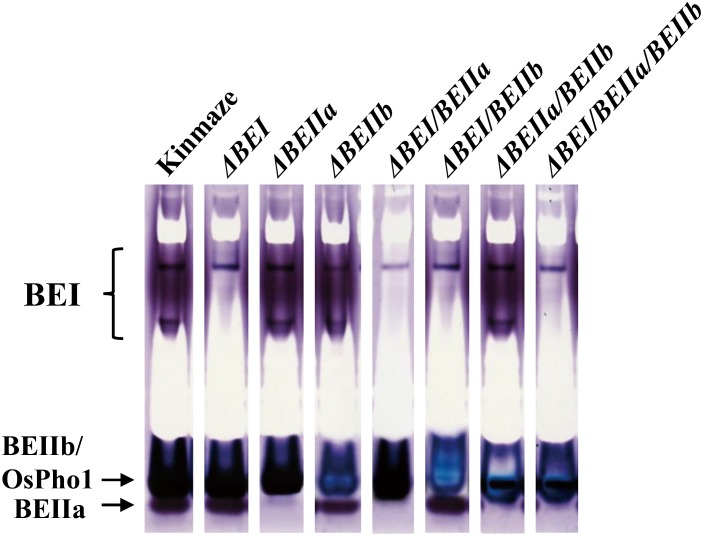
Native-PAGE/activity staining of BEs in crude enzyme extracts from developing kernels of the transformed lines, wild-type (cv. Kinmaze), and the *be2b* mutant line, EM10. The developing T_1_ kernels of transformed lines from which the enzyme extracts were prepared are (from left to right): #2 for Δ*BEI*; #8 for Δ*BEIIa*; #6 for Δ*BEIIb*; #11 for Δ*BEI/BEIIa*; #5 for Δ*BEI/BEIIb*; #11 for Δ*BEIIa/BEIIb*; and #9 for Δ*BEI/BEIIa/BEIIb*. Note that in the BEIIb suppression lines, the BEIIb-corresponding bands exhibited a blue color, which was due to the activity of plastidial phosphorylase 1 (Pho1) only present in rice endosperm, while in the other lines, these bands exhibited dark reddish purple colors, indicating the presence of branched glucans formed by BEIIb and Pho-a as well as Pho1.

Out of about 30 lines regenerated from each RNAi construct, 6–9 in which enzyme activities were suppressed as assessed by zymogram (Figure 2) were selected: #1, 2, 8, 12, 14, 19, and 25 for Δ*BEI* lines; #2, 4, 5, 6, 8, 10, and 14 for Δ*BEIIa* lines; #2, 5, 6, 8, 12, 13, 15, and 27 for Δ*BEIIb* lines; #3, 11, 14, 17, 27, and 29 for Δ*BEI/BEIIa* lines; #2, 5, 6, 9, 11, 13, 16, 22, and 27 for Δ*BEI/BEIIb* lines; #4, 6, 7, 11, 12, and 27 for Δ*BEIIa/BEIIb* lines; and #4, 7, 9, 12, 24, and 28 for Δ*BEI/BEIIa/BEIIb* lines. Since T_1_ seeds were unable to germinate, biochemical analyses were performed using T_1_ seeds generated from one of the representative T_0_ progeny lines exhibiting the most similar kernel morphology and starch-related phenotypes among them in each construct.

### BE Isozyme Activity Levels in Transformed Lines

To examine to what extent BE isozyme activities were suppressed in the transformed lines by the RNAi constructs, the crude enzyme extracts prepared from developing kernels were separated by native PAGE to determine the activities of BEI, BEIIa, and BEIIb by an activity-staining method. BEIIb activity band is known to overlap with Pho1 band on the native PAGE (Yamanouchi and Nakamura, 1992; Nishi et al., 2001). When BEIIb activity is high, these bands are a reddish purple color due to the formation of branched glucans. However, when BEIIb activity is lost, the band color turns blue because only linear glucans are synthesized by Pho1 in the presence of glucose 1-phosphate (Yamanouchi and Nakamura, 1992). Figure 2 shows that all the BE activities were greatly reduced in endosperms from the representative lines (#2 for Δ*BEI* line; #8 for Δ*BEIIa* line; #6 for Δ*BEIIb* line; #11 for Δ*BEI/BEIIa* line; #5 for Δ*BEI/BEIIb* line; #11 for Δ*BEIIa/BEIIb* line; and #9 for Δ*BEI/BEIIa/BEIIb* line). Although a small amount of BEI activity remained in the Δ*BEI*-related transformants, almost all of BEIIa activity was lost in the Δ*BEIIa*-related transformants. BEIIb activity was largely suppressed in Δ*BEIIb*-related transformants. Together, these results show that all the RNAi constructs, for the most part, successfully silenced the appropriate *BE* genes, as they were designed to.

### Transformed Line Kernel Morphology

**FIGURE 3:**
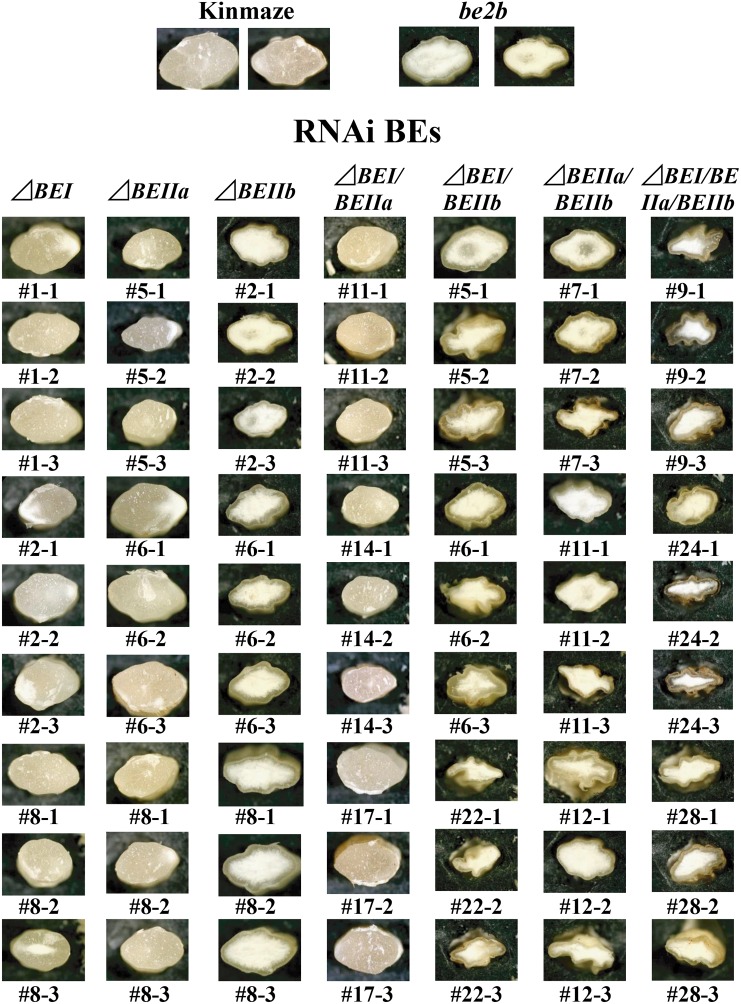
Light micrographs of cross-sections of mature kernels from the transformants, their host wild-type *japonica* cultivar Kinmaze, and a *be2b* mutant line, EM10.

Three lines each for every construct were chosen from those having kernels of the consistent size and morphology, as shown in Figure 3.

Wild-type cultivars of *japonica*-type rice have the plump seeds with translucent kernels, whereas some mutants, such as *ae* mutant, have a floury kernel phenotype (Nishi et al., 2001; Nakamura, 2018). The Δ*BEI*, Δ*BEIIa*, and Δ*BEI/BEIIa* lines had plump translucent kernels, and their appearances were the same as the wild-type (Figure 3). The Δ*BEIIb* kernels had a floury kernel phenotype that were not shriveled, although their size was somewhat smaller than the wild-type (Figure 3). This phenotype was the same that of the *ae* mutant line, EM10 (Figure 3). However, most of kernels from the double and triple Δ*BEIIb*-related transformants such as Δ*BEIIb/BEIIa*, Δ*BEIIb/BEIIa*, and Δ*BEIIb/BEIIa/BEI* were severely shrunken and had a floury phenotype (Figure 3).

One representative line was chosen from the three selected T_0_ progeny lines: #2 for Δ*BEI*; #8 for Δ*BEIIa*; #6 for Δ*BEIIb*; #11 for Δ*BEI/BEIIa*; #5 for Δ*BEI/BEIIb*; #11 for Δ*BEIIa/BEIIb*; and #9 for Δ*BEI/BEIIa/BEIIb*. A single T_1_ kernel was chosen from these lines, cut in half, and each half used for analysis of starch thermal properties or amylopectin chain-length distribution so that the relationship between amylopectin fine structure and thermal properties of starch granules could be assessed.

### Mature Kernel Weights of Transformed Lines

**FIGURE 4:**
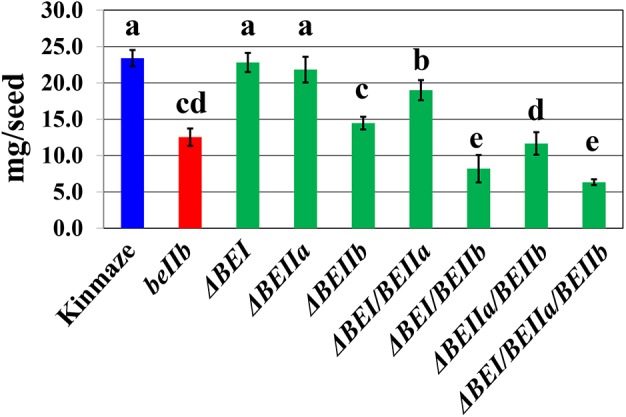
The average weight of a single kernel in each transformed line, the host wild-type *japonica* cultivar Kinmaze, or a *be2b* mutant line, EM10. Values are the averages measured from 15 arbitrarily chosen mature caryopses and standard deviations are presented. The lines used were the same as those in Figure 3 and Supplementary Figure S2. Values indicated by the same letter above the column are not significantly different as calculated by the Tukey–Kramer method (*p* < 0.05).

The *be2b* mutant of *japonica*-type rice has a reduced kernel weight (Nishi et al., 2001), while that of the *be1* mutant is similar to wild-type (Satoh et al., 2003). The kernel weights of the Δ*BEI* or the Δ*BEIIa* transformant was similar or slightly lower than wild-type (Figure 4 and Supplementary Figure S2). However, the kernel weight of the Δ*BEIIb* line was significantly lower than that of wild-type, and similar to a *be2b* mutant line EM10 (Figure 4 and Supplementary Figure S2). Although kernel weight was unaffected by silencing of the *BEIIa* or *BEI* gene, it was significantly lower in the Δ*BEI/BEIIa* line than that in the Δ*BEI* or Δ*BEIIa* line (Figure 4 and Supplementary Figure S2). The kernel weight of the Δ*BEIIa/BEIIb* line was significantly lower than that of the Δ*BEIIb* line (Figure 4 and Supplementary Figure S2). These results show that BEIIa plays a role in amylopectin biosynthesis in the absence of BEI or BEIIb. Similarly, even though the Δ*BEI* line had the same kernel weight as the wild-type, the kernel weight of the Δ*BEI/BEIIb* line was markedly decreased and significantly lower than that of the Δ*BEIIa/BEIIb* line (Figure 4 and Supplementary Figure S2). These results indicate that the BEI’s role in amylopectin synthesis is more important than that of BEIIa, particularly when BEIIb activity is very low or missing. The kernel weight of the Δ*BEI/BEIIa/BEIIb* line was similar to the Δ*BEI/BEIIb* line and the lowest among all of the suppression lines (Figure 4). These results suggest that the residual activities of all the three BE isozymes were too low to support starch biosynthesis in rice endosperm, and that any BE can support synthetic activity, at least to some extent, especially when any of the other isozymes are defective.

### Chain-Length Distribution of Amylopectin of Transformed Line Kernels

To examine the contribution of each BE isozyme to the fine structure of amylopectin, we determined the chain-length distribution of amylopectin after debranching the insoluble glucans with PaISA followed by labeling with APTS at their non-reducing ends, according to the FACE method (O’Shea et al., 1998).

**FIGURE 5:**
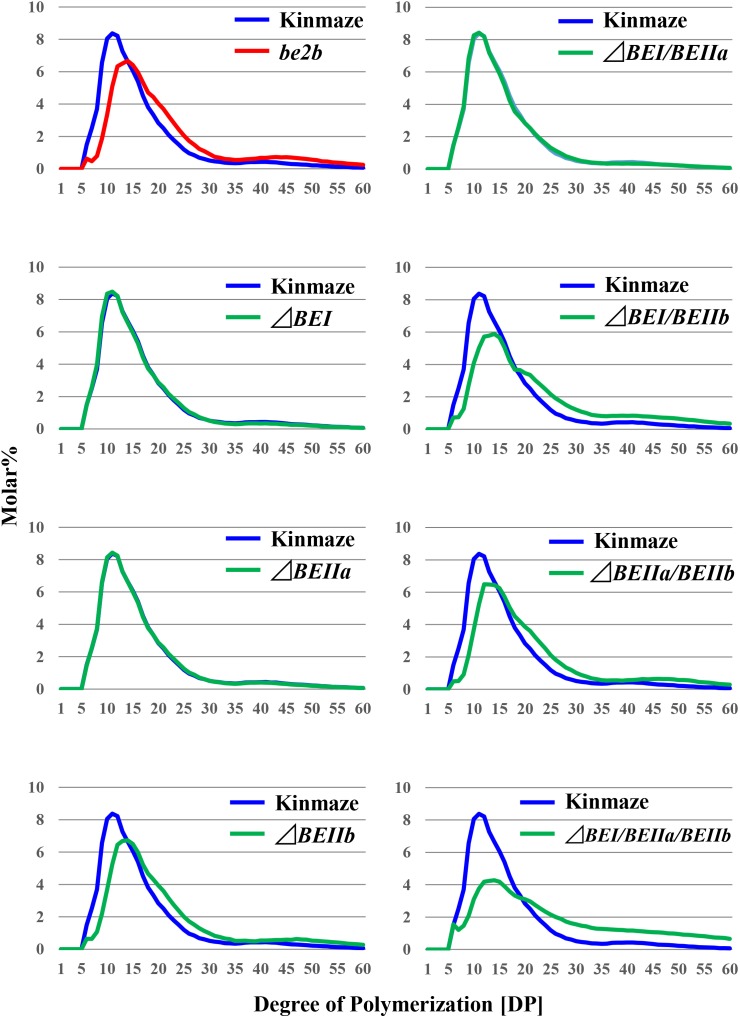
Chain-length distribution of amylopectin in mature endosperm from the rice transformants, their host cultivar Kinmaze, or the *be2b* mutant line, EM10. Values are the averages calculated from three replicate measurements. Standard deviations were too small to be shown in the figure. The mature T_1_ kernels of transformed lines from which starches were prepared are (from left to right): #2 for Δ*BEI*; #8 for Δ*BEIIa*; #6 for Δ*BEIIb*; #11 for Δ*BEI/BEIIa*; #5 for Δ*BEI/BEIIb*; #11 for Δ*BEIIa/BEIIb*; and #9 for Δ*BEI/BEIIa/BEIIb*.

**FIGURE 6:**
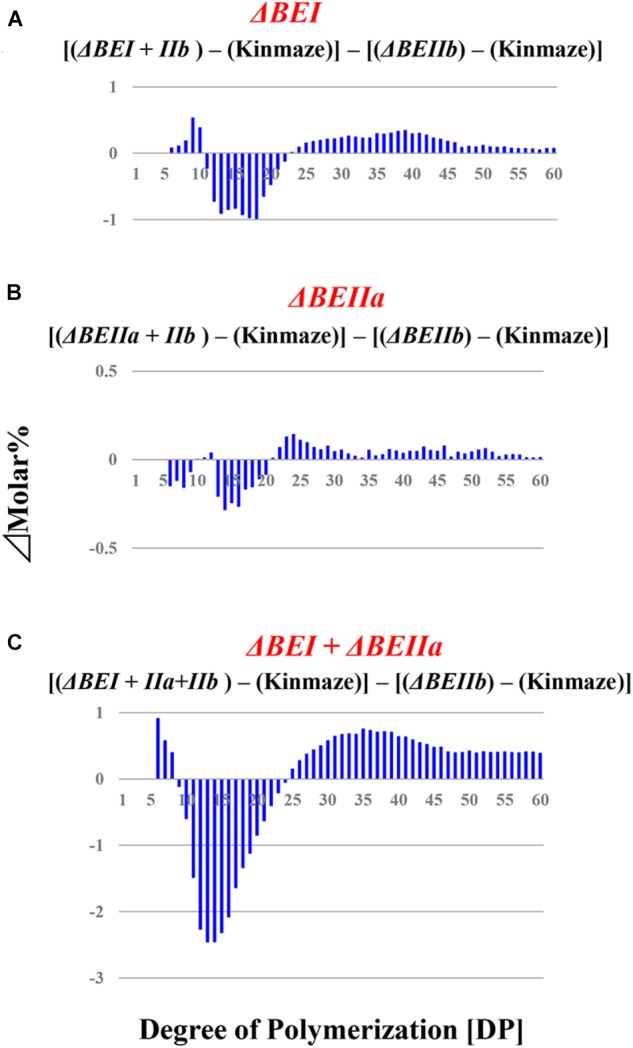
Difference in chain-length distributions of amylopectin from each transformant compared to Δ*BEIIb*, as shown in figures **(A–C)**. Data are the same as those shown in Figure 5.

The chain profiles of amylopectin produced in endosperm of the Δ*BEI* or Δ*BEIIa* line was very similar to that from wild-type (Figures 5, 6), suggesting that the contributions of BEI and BEIIa to amylopectin fine structure are unspecific and that, in the absence of either isozyme, the remaining BE isozymes can complement their functions. Our previous study indicated that amylopectin in the *be1* mutant contains more DP ≤ 10 chains and fewer DP ≥ 37 and DP12-21 chains, although the extents of these changes are much less significant compared with those in the *be2b* mutant (Nishi et al., 2001; Nakamura, 2002, 2015; Satoh et al., 2003). The Δ*BEI* line still contained a small amount of BEI activity (Figure 3), suggesting that the apparent discrepancy between the *be1* mutant and the Δ*BEI* line was due to the difference in the residual BEI activity level between them; i.e., none or extremely low in the mutant versus very low but still at a functional level in the transformant. In contrast, amylopectin in the Δ*BEIIb* line had fewer short chains of DP ≤ 13 with a peak at DP9 and 10 and more intermediate B1 and long B2-3 chains than in the wild-type; these changes were consistent with those in the *be2b* (*ae*) mutant (Figure 5 and Supplementary Figures S1, S3) (Nishi et al., 2001).

When the expression of both *BEI* and *BEIIb* genes was simultaneously suppressed, as in the Δ*BEI/BEIIb* line, the crosspoint of the difference graph shifted from DP13/14 in the Δ*BEIIb* line to DP18/19 in the Δ*BEI/BEIIb* line (Supplementary Figure S3). In addition, long chains with DP ≥ 30 were significantly higher in the Δ*BEI/BEIIb* line compared to Δ*BEIIb*. This strongly suggests that BEI plays an important role in the synthesis of a wide range of longer A chains with DP up to 14–18 and B chain external segments with chain-lengths up to DP18, while BEIIb is only involved in the synthesis of short A chains and short B chain external segments with DP ≤ 13. On the other hand, the chain-length distribution pattern of amylopectin in the Δ*BEIIa/BEIIb* line was almost the same as in Δ*BEIIb* (Supplementary Figure S3), indicating that BEIIa cannot compensate for the role of BEIIb in amylopectin synthesis. Interestingly, however, amylopectin in the Δ*BEI/BEIIa/BEIIb* line had fewer chains of DP6-18 and more chains of DP ≥ 30 compared with Δ*BEI/BEIIb* (Supplementary Figure S3). One simple explanation of these results is that BEIIa can complement, at least to some extent, the role of BEI rather than that of BEIIb.

### Thermal Properties of Starch in Transformed Line Kernels

The thermal properties of starch granules in various BE transformants were compared with those in wild-type or the *ae* (*be2b*) mutant using DSC (Table 1). As reported previously (Nishi et al., 2001), the *be2b* mutation caused starch granules to be highly resistant to thermal gelatinization (Table 1). In accordance with this result, the starch granules in the Δ*BEIIb* line had markedly higher DSC parameters compared with wild-type starch granules (Table 1). It is interesting that the DSC parameters of the Δ*BEIIa* starch granules were slightly higher than those of wild-type (Table 1), although the chain-length distribution of amylopectin in the Δ*BEIIa* line was similar to that in the wild-type, as described above (Supplementary Figure S3). It is also notable that the starch granules were much more resistant to thermal gelatinization when expression of both *BEIIa* and *BEIIb* genes was suppressed (Table 1). In contrast, the thermal properties of starch granules were unchanged by suppression of the *BEI* gene expression. Together, these results suggest that BEIIa plays some role in the formation of starch granule structure despite its direct contribution to chain-length distribution being seemingly low.

**Table 1:** Thermal properties of native starches from mature kernels of Δ*BE*-transformed lines, their host wild-type *japonica* cultivar Kinmaze, and a *be2b* mutant line EM10.

Line	To (°C)	Tp (°C)	Tc (°C)	ΔH (J/g)
Kinmaze	53.7 ± 0.7	59.3 ± 0.9	65.0 ± 0.9	4.0 ± 1.0
*be2b*	60.1 ± 0.8	74.6 ± 0.6	82.8 ± 0.8	5.6 ± 0.9
Δ*BEI*	54.7 ± 1.6	62.3 ± 1.8	67.6 ± 2.3	5.2 ± 1.9
Δ*BEIIa*	56.2 ± 0.3	64.6 ± 1.4	70.4 ± 0.5	5.8 ± 1.1
Δ*BEIIb*	61.1 ± 0.7	75.7 ± 0.3	84.1 ± 1.4	7.1 ± 1.6
Δ*BEI/BEIIa*	53.2 ± 0.7	60.4 ± 0.6	66.8 ± 0.6	4.7 ± 0.9
Δ*BEI/BEIIb*	nd			
Δ*BEIIa/BEIIb*	67.0 ± 0.6	77.3 ± 0.4	83.1 ± 0.1	6.3 ± 0.2
Δ*BEI/BEIIa/BEIIb*	nd			

*T_0_, Tp, Tc, and ΔH are onset, peak, conclusion temperature, and enthalpy change, respectively. Values are the averages calculated from three replicate measurements with standard deviations. nd, not determined. In this study, we focused on the relationship between the chain-length distribution of amylopectin and the thermal properties of starch granules from the same kernel. However, starch contents of the kernels from ΔBEI/BEIIb and ΔBEIIa/BEIIb lines were too low to measure by DSC.*

### Role of Each BE Isozyme in Amylopectin Biosynthesis

We have performed a detailed analysis of the changes in the chain-length distribution of amylopectin produced in rice endosperm when the activities of a single or all the possible combinations of the three BE isozymes were inhibited. The pattern of changes largely depended on the BE isozyme(s) that was (were) deficient in the endosperm. To ascertain clearly the contribution of BEI and BEIIa to the amylopectin fine structure, the differences between the chain-length distribution of amylopectin between the Δ*BEIIb* line and the Δ*BEI/BEIIb*, Δ*BEIIa/BEIIb*, or Δ*BEI/BEIIa/BEIIb* line was compared (Figure 6), because this might clarify the additional effect of the reduction of BEI activity and/or BEIIa activity.

The contribution of BEI to amylopectin synthesis could be conceived from the difference of the chain-length pattern between the Δ*BEI/BEIIb* line and the Δ*BEIIb* line (Figure 6A). The intermediate DP11-21 chains of amylopectin from the Δ*BEI/BEIIb* line were significantly lower than those from the Δ*BEIIb* line, indicating that BEI plays a distinct role in the synthesis of the intermediate chains. It is interesting to note that intermediate chains of DP13-20 were also slightly reduced in the Δ*BEIIa/BEIIb* line compared with the *BEIIb* line (Figure 6B). This suggests that BEIIa plays a part in the synthesis of these chains. The amylopectin from the Δ*BEI/BEIIa/BEIIb* line had markedly fewer intermediate chains of DP11-22 than the Δ*BEIIb* line (Figure 6C), and the difference in amylopectin chain-length distribution between the Δ*BEI/BEIIa/BEIIb* and Δ*BEIIb* lines was much larger than that between the Δ*BEI/BEIIb* and Δ*BEIIb* lines (c.f. Figure 6C with Figure 6B). These results also support the view that both BEI and BEIIa play an important role in the synthesis of intermediate chains. Thus, a distinct role of BEIIa in amylopectin synthesis in rice endosperm has been assigned for the first time in this study, because it was impossible to determine the contribution of BEIIa to the amylopectin structure in the past since no significant effect of BEIIa on the amylopectin fine structure was observed in the *be2a* mutant (Nakamura, 2002).

### Comparison of the Roles of BE Isozymes Among Cereal Endosperms

There have been numerous studies that have examined the roles of BE isozymes in endosperm of cereals such as maize, rice, wheat, and barley (see the review by Nakamura, 2015; Tetlow and Emes, 2017). The impact of the loss of each BE isozyme on endosperm starch structure has been reported to be similar between maize and rice. A deficiency of either BEI or BEIIa results in no detectable or only small changes in endosperm starch phenotypes and kernel morphology in maize and rice (Blauth et al., 2001, 2002; Nakamura, 2002; Satoh et al., 2003; Yao et al., 2004). In contrast, a number of groups worldwide have reported on the specific impact of BEIIb on the fine structure, starch granule morphology, and starch physicochemical properties in endosperms from maize (Yuan et al., 1993; Shi and Seib, 1995; Klucinec and Thompson, 2002; Yao et al., 2004; Li et al., 2007) and rice (Nishi et al., 2001; Tanaka et al., 2004; Wei et al., 2010; Butardo et al., 2011; see also reviews by Wang et al., 2017; Nakamura, 2018).

It is interesting that the effects of BEII isozyme inhibition on starch structure and properties seem to differ largely among four major crops: maize, rice, wheat, and barley. Regina and her colleagues have revealed that suppression of BEIIa expression greatly affects starch structure, amylose content, and starch physicochemical and functional properties of starch granules in wheat and barley endosperm, and its influences are more severe than the loss of BEIIb activity (Regina et al., 2006, 2010, 2015). The results contrast strikingly with the observation that a single mutation of BEIIa produces no significant effect on starch structure and properties in both maize (Blauth et al., 2001) and rice (Nakamura, 2002) (Figure 5) endosperm. It is known that BEIIb is present at lower levels in wheat endosperm than that in maize and rice endosperm (Regina et al., 2005). On the other hand, the observation that starch structure and properties are largely controlled by varying BEIIb levels under constant BEIIa levels in rice endosperm (Tanaka et al., 2004) clearly indicates that these changes in starch are caused by a specific role of BEIIb, but not BEIIa, in amylopectin biosynthesis. The molecular mechanism for the discrepancy in the functions of BEIIa and BEIIb found among cereals remains to be elucidated in the future.

## Conclusion

In the present study, the contributions of all three BE isozymes, namely BEI, BEIIa, and BEIIb, to amylopectin biosynthesis in rice endosperm were comprehensively examined by analyzing amylopectin chain-length distribution in seven lines in which all the combinations of BE expressions were singly or multiply silenced with RNAi. Thus, the present study could clarify the distinct and overlapping roles of these individual isozymes. Lack of BEIIb led to the most striking changes in starch-related phenotypes in rice endosperm, indicating that this isozyme plays a crucial role in the starch biosynthesis, particularly, in the formation of amylopectin short chains. These results are consistent with *in vivo* studies with BEIIb suppressed lines (Wei et al., 2010; Butardo et al., 2011), and *in vitro* experiments with purified enzyme, showing that BEIIb forms external short chains of DP7 and 6 (Nakamura et al., 2010).

A previous study with a rice *be1* mutant suggested that BEI is involved in the synthesis of intermediate amylopectin chains (Satoh et al., 2003). However, its role was unclear because the chain-length of amylopectin was only slightly altered by the *be1* mutation. In the present study, the role of BEI could be identified by comparing all the BEI-related transformants (Figures 5, 6 and Supplementary Figure S3). Figure 6 distinctly indicates that BEI is involved in the synthesis of intermediate amylopectin chains.

Up to now, no distinct role of BEIIa in developing rice endosperm has been proposed because no significant change in amylopectin chain-profile was detected in the *be2a* mutant (Nakamura, 2002), as also seen in the BEIIa-suppressed line (Figure 5 and Supplementary Figure S3). The present study, however, shows that BEIIa plays a substantial role in starch biosynthesis in rice endosperm. Detailed analysis of the chain-length distribution of amylopectin clearly indicates that BEIIa compensates for role of BEI, rather than BEIIb, by forming intermediate chains (Figure 6). The result is rather surprising in terms of amino acid sequence homology among three BE isozymes, because BEIIa is more alike to BEIIb, rather than BEI (Sawada et al., 2014). However, the result seems to be consistent with our previous report that the amylopectin chain-profile was only controlled by levels of BEIIb activity when BEIIa levels were constant, and therefore it was unaffected by relative activities of BEIIa and BEIIb (Tanaka et al., 2004). The additional striking observation in the present study was that loss of BEIIa resulted in an increase in the onset temperature of thermal gelatinization (T_0_), and this effect was more pronounced in the Δ*BEIIa/BEIIb* line (Table 1). The results strongly suggest that BEIIa is more significantly involved in the construction of starch granules than previously appreciated.

In summary, the present investigation provides concrete evidence on the distinct contributions of three BE isozymes to the synthesis of amylopectin fine structure, the formation of starch granules, and starch properties in rice endosperm.

## Author Contributions

TS and MI conducted experiments. TS also summarized data and prepared Figures and Table. YN designed the experiments and wrote the paper.

## Conflict of Interest Statement

The authors declare that the research was conducted in the absence of any commercial or financial relationships that could be construed as a potential conflict of interest.
