# Supplementary figures and images for: Contributions of Three Starch Branching Enzyme Isozymes to the Fine Structure of Amylopectin in Rice Endosperm
Source: Front Plant Sci. 2018 Oct 23;9:1536. doi: 10.3389/fpls.2018.01536 (PMC6206275; doi:10.3389/fpls.2018.01536)

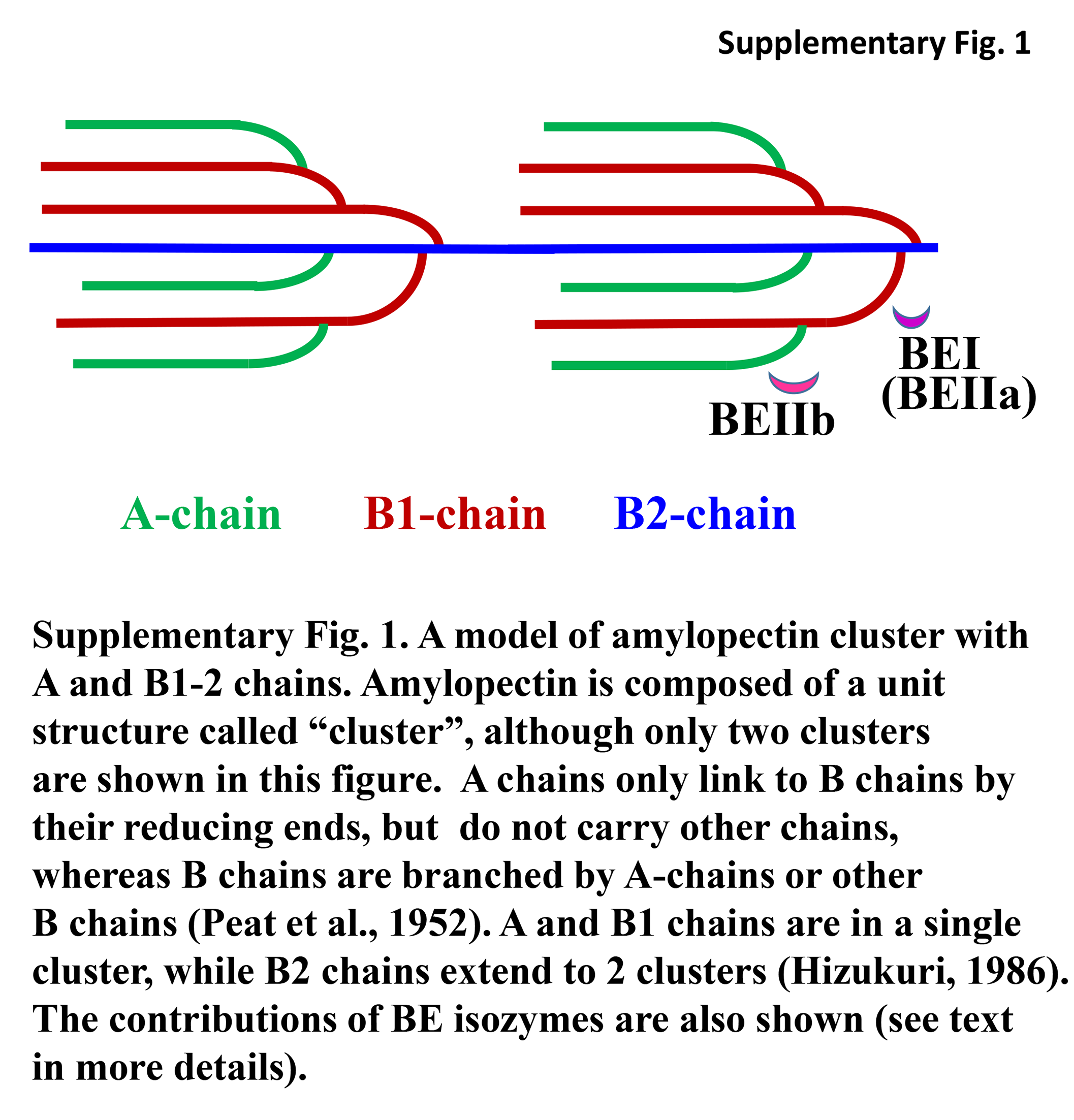

Supplement: Supplementary file 1 [file Image_1.TIF]

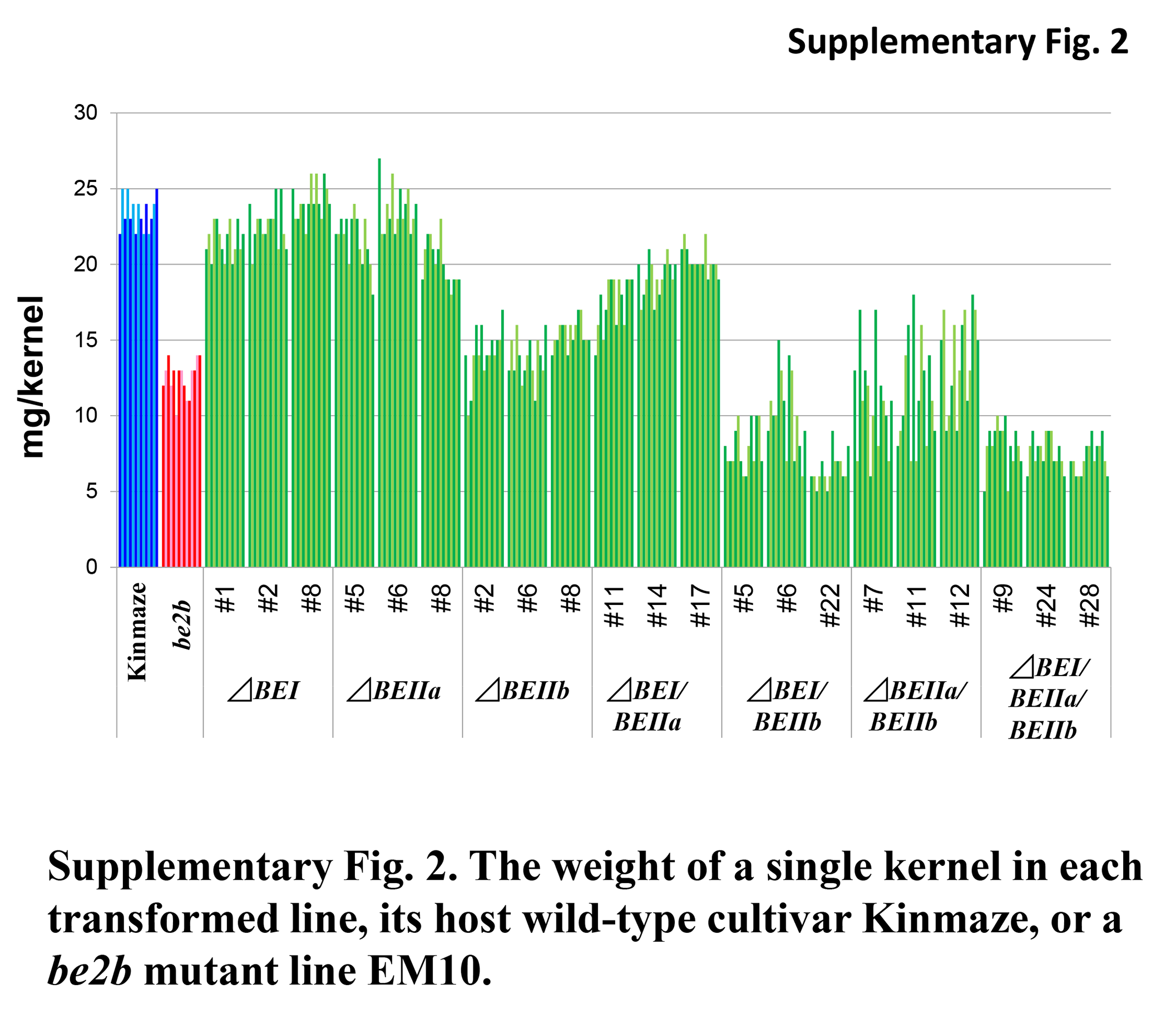

Supplement: Supplementary file 2 [file Image_2.TIF]

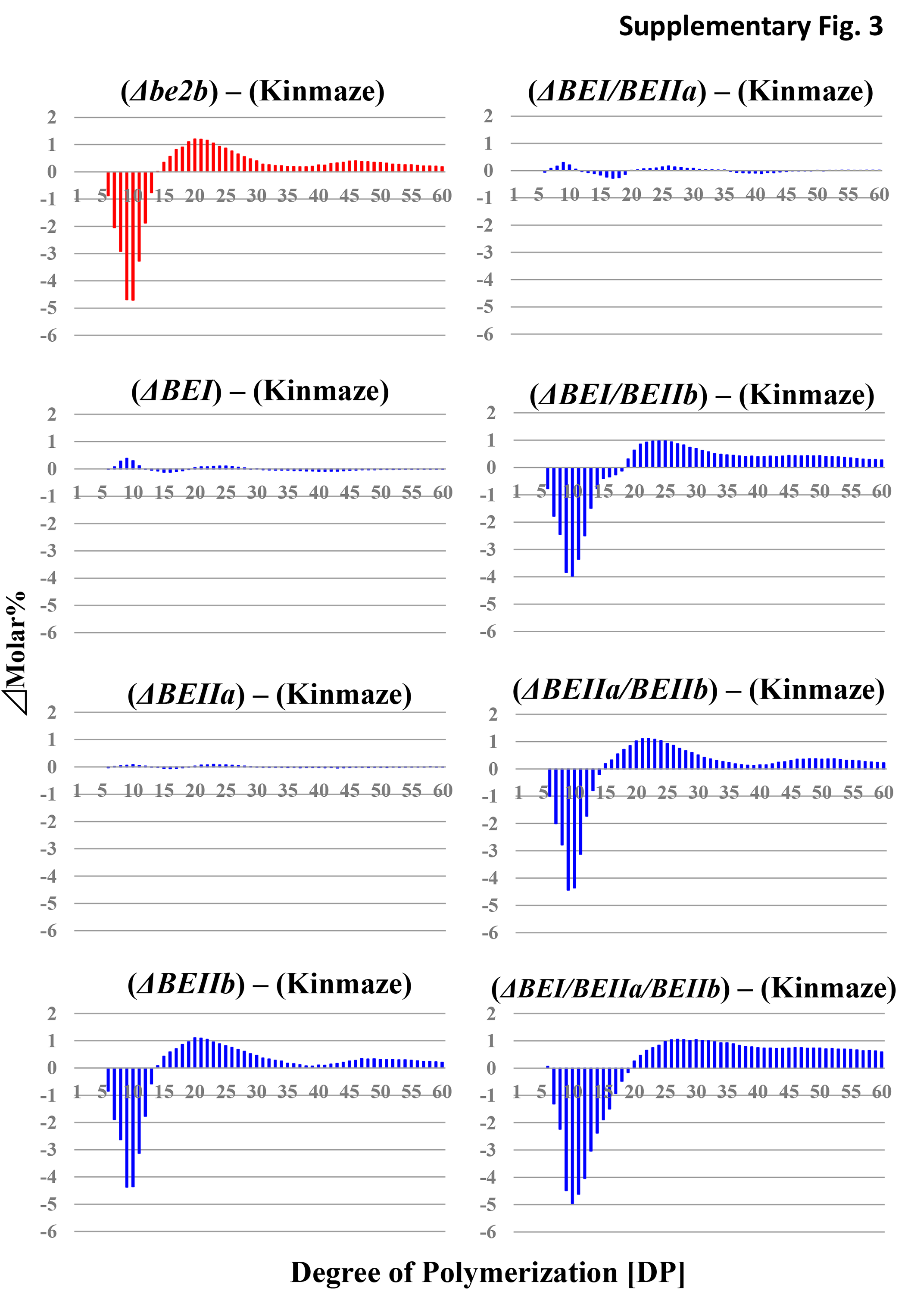

Supplement: Supplementary file 3 [file Image_3.TIF]

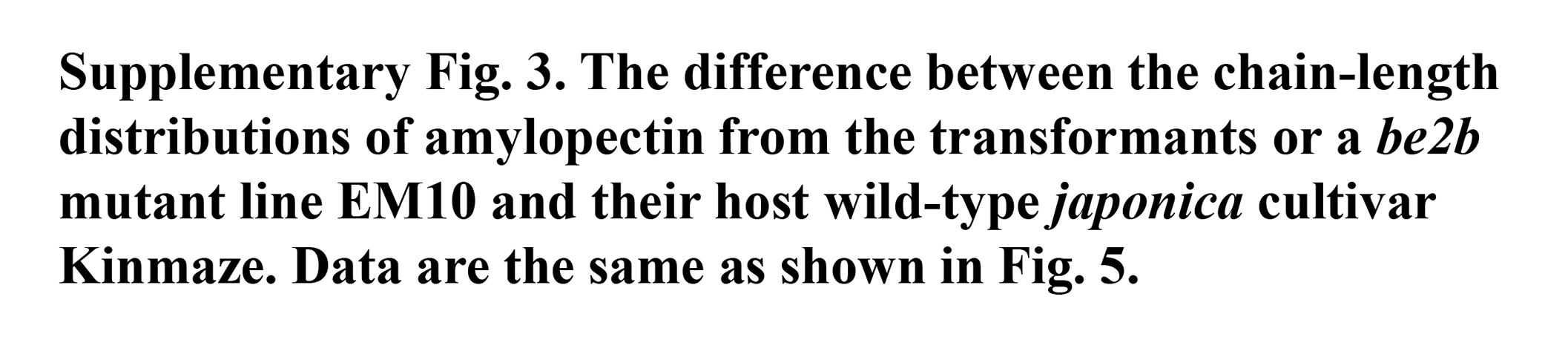

Supplement: Supplementary file 4 [file Image_4.TIF]
